# 

**Published:** 1882-10

**Authors:** 


### TABLE OF CONTENTS FOR OCTOBER, 1882.

### ORIGINAL COMMUNICATIONS.

PAGE.
ART. I.—A Winter Cold. By F. H. BOSWORTH, M. D .................. 561
ART. II.—An Incident of Practice. By C. E. FRANCIS, D.D.S., M.D.S... 567

ORIGINAL TRANSLATIONS AND ABSTRACTS.

Ergot in the Treatment of Glycosuria.............................. 568
The Subcutaneous Injection of Ether.............................. 569
The American Academy of Dental Science.......................... 569

SELECTIONS.

Abstract of Paper on Operation for Closure of the Hard Palate and Hare-Lip immediately after Birth.............................. 570
Crural Neuralgia among Dentists.................................. 572
Oleoresin of Male Fern : Increasing its Efficacy against Tapeworm......... 573
Guachamaca Extract.............................................. 574
Remarks of Dr. W. C. Barrett upon Dental Literature................ 575
Extraction of Teeth of Pregnant Women............................ 578
Traumatic Tetanus treated with Eserine........................... 579
Aconite in Dysentery ............................................ 579
Impotency....................................................... 580
Proprietary Medicines........................................... 580
The Opium Habit................................................. 580
CURRENT NEWS AND OPINION........................................ 582
BIBLIOGRAPHICAL ................................................ 583
POPULAR SCIENCE DEPARTMENT...................................... 586

